# Genetic diversity and population structure of rice landraces from Eastern and North Eastern States of India

**DOI:** 10.1186/1471-2156-14-71

**Published:** 2013-08-15

**Authors:** Basabdatta Das, Samik Sengupta, Swarup Kumar Parida, Bipasha Roy, Mrityunjay Ghosh, Manoj Prasad, Tapas Kumar Ghose

**Affiliations:** 1 Division of Plant Biology, Bose Institute, Main Campus, 93/1 A.P.C. Road, Kolkata, West Bengal, 700009, India; 2 Department of Horticulture, Institute of Agricultural Science, University of Calcutta, 35, Balligunge Circular Road, Kolkata, West Bengal, 700029, India; 3 Department of Agronomy, Bidhan Chandra Krishi Viswavidyala Mohanpur, District – Nadia – 741252, Kolkata, West Bengal, India; 4 National Institute of Plant Genome Research (NIPGR), Aruna Asaf Ali Marg, New Delhi, 110067, India

**Keywords:** *Oryza sativa*, Landrace, Genetic diversity, SSR polymorphism, Population structure

## Abstract

**Background:**

Adaptations to different habitats across the globe and consequent genetic variation within rice have resulted in more than 120,000 diverse accessions including landraces, which are vital genetic resources for agronomic and quality traits. In India the rice landraces of the states West Bengal, Assam, Mizoram, Manipur and Nagaland are worthy candidates for genetic assessment. Keeping the above in view, the present study was conducted with the aim to (i) calculate the genetic distances among the accessions of 83 landraces collected from these states along with 8 check accessions (total 91 accessions) using 23 previously mapped SSR markers and (ii) examine the population structure among the accessions using model-based clustering approach.

**Results:**

Among the 91 accessions, 182 alleles were identified which included 51 rare and 27 null alleles. The average PIC value was 0.7467/marker. The non-aromatic landraces from West Bengal was most diverse with 154 alleles and an average PIC value of 0.8005/marker, followed by the aromatic landraces from West Bengal with 118 alleles and an average PIC value of 0.6524/marker, while the landraces from North East ranked third with 113 alleles and an average PIC value of 0.5745/marker. In the dendrogram distinct clusters consisting of predominantly aromatic landraces and predominantly North East Indian landraces were observed. The non-aromatic landraces from West Bengal were interspersed within these two clusters. The accessions were moderately structured, showing four sub-populations (A-D) with an *Fst* value of 0.398, 0.364, 0.206 and 0.281, respectively. The assigned clustering of accessions was well in agreement in both distance-based and model-based approaches.

**Conclusions:**

Each of the accessions could be identified unequivocally by the SSR profiles. Genetically the non aromatic landraces from West Bengal were most diverse followed by the aromatic landraces from the same state. The North Eastern accessions ranked third. Further, grouping of accessions based on their agronomic traits may serve as a resource for future studies, leading to the improvement of rice. Moreover in-situ preservation of the landraces is also a means of protection of biodiversity and cultural heritage.

## Background

Rice (*Oryza sativa* L.) is a major ingredient in cuisines world over, in the form of breakfast cereals, staple carbohydrate, snacks, alcoholic beverage and desserts. In addition to the two major subspecies, japonica and indica, [1] several other minor rice types have been identified with genetic markers [2,3] which include the upland drought-tolerant Aus germplasms of India and Bangladesh, the deep-water Ashinas of Bangladesh, and the aromatic Basmati rice of India. As a consequence of adaptations to different habitats, extensive genotypic and phenotypic diversity exists within *O*. *sativa*, resulting in about 120,000 different accessions [4]. These accessions range from traditional rice landraces preserved by indigenous farmers to the commercially bred cultivars developed during the green revolution. According to Harlan [5] landraces are “balanced populations in equilibrium with both the environment and pathogens, and are genetically dynamic”. They are local varieties of a domesticated plant species which were adapted to the natural and cultural environment in which they live. Each landrace has particular properties or characteristics; early maturity, adaptation to particular soil types, resistance or tolerance to biotic and abiotic stresses, and in the expected end usage of the grains. India is home to many such landraces and the ones from the state of West Bengal and North Eastern States of the country are especially diverse morphologically and genetically, and are worthy candidates for detailed examination. This study estimates a broad overview of Simple Sequence Repeat (SSR) based genetic diversity present in 83 different rice landrace selected from the wide array available in the states of West Bengal, Assam, Nagaland, Mizoram and Manipur using 23 SSR markers.

The topography of the state of West Bengal and the seven North Eastern States of India namely, Arunachal Pradesh, Assam, Manipur, Meghalaya, Mizoram, Nagaland and Tripura, is one of nature’s marvels which has brought in proximity the snow capped peaks of the Himalayas, the ecological hot spots of the North Eastern foothills, the Brahmaputra valley, the fertile Gangetic plain, and the estuarine regions of the Sunderban delta. High rainfall, humidity, varied topography and altitude, heavy natural selection pressures of diseases and pests, introductions over time and space from adjoining countries, introgression from the wild and weedy relatives, tribal preferences and environmental stresses have made the region rich both in floristic and crop diversities [6].

Molecular markers, especially DNA-based markers, have been used extensively for the study of genetic diversity, unambiguous identification of germplasm and their protection under the trade related intellectual property rights (TRIPS) of the World Trade Organization (WTO). Mackill [7] classified 134 japonica rice cultivars (both traditional and modern) using RAPD and was successful in separating the temperate and tropical japonica genotypes. Glaszmann [8] quantified the genetic diversity of 289 rice cultivars from various parts of Northeast India at 14 isozyme loci and was able to segregate the cultivars into the varietal groups I to VI. The gene diversity index [9] for that study was 0.341 for Northeast Indian rice which was close to that calculated for all Asian rice (0.346), and thus very high considering the small size of the area under survey. SSR or Simple Sequence Length Polymorphism (SSLP) markers have been used for evaluating rice genetic diversity by a number of groups [10-14] for estimating variation between the indica and japonica subspecies [15] and classifying *Oryza sativa* L. genotypes [16]. Blair *et al*., [17] differentiated indica and japonica rice genotypes using Inter Simple Sequence Repeat (ISSR) polymorphism data. ISSR polymorphism has also been used for genetic diversity and phylogenetic analysis in 42 genotypes including 17 wild *Oryza* species [18]. ISSR and SSR markers have been used to study genetic diversity of *Oryza nivara* Sharma *et* Shastry, genotypes collected from different geographical regions [19]. Herrera *et al*. [20] used 48 simple-sequence-repeat (SSR) markers to assess the genetic diversity of 11 Venezuelan rice cultivars released by the National Rice Breeding Program between 1978 and 2007 and detected 203 alleles. Pervaiz *et al*., [21] used 35 SSR markers to detect genetic diversity in 75 rice landraces and identified 142 alleles. The polymorphism information content (PIC) ranged from 0.124 to 0.836, with an average of 0.569. Maytinee *et al*., [22] used InDel (Insertion/Deletion), ISSR and SSR markers to detect genetic diversity among 126 Thai rice accessions. Behera *et al*., [23] used 36 microsatellite markers to assess genetic diversity in a set of 33 medicinal rice genotypes and detected 166 polymorphic loci. The PIC values ranged between 0.24 and 0.956 with an average of 0.811 per locus. However a thorough SSR based genetic diversity analysis, of the landraces of West Bengal and North Eastern States is yet to be undertaken.

Previous workers like Ge *et al*., [24] have reported the linkage of SSR loci on rice chromosome 2 with cooked kernel expansion and the same for chromosome 3 with width expansion of rice kernels. Quantitative Trait Loci (QTL) mapping of rice by Bai *et al*., [25] have indicated the linkage of rice grain shape (grain length/ grain breadth ratio) to SSR loci RM112 and RM530 on chromosome 2. Ahmadi and Fotokian [26] have indicated the linkage of markers RM251 and RM282 on rice chromosome 3 to potassium ion concertration. Lin *et al*., [27] have shown linkage of RM112 and RM530 on chromosome 2 to leaf rolling in rice plants due to water stress. QTL mapping of rice indicated the linkage of rice grain and kernel length, breadth, length/breadth ratio kernel length after cooking and aroma to 18 different SSR markers [28,29]. The set of markers used in this study to assess genetic diversity included 18 markers from the references above along with 5 more (a total of 23 markers) with linkage to Sheath blight and Blast resistance [30] HSP 80 gene [31] and Beta amylase gene. Only the most popular landraces that are regularly cultivated by small farming communities were chosen for this study. Each of the landraces had some special characteristic like aroma, disease resistance, yield etc for which they have been cultivated by farmers down the ages. Keeping the above in view, the present study was conducted with the aim to (i) calculate the genetic distances among 91 rice germplasm using 23 mapped SSR markers; and (ii) examine the population structure among the accessions using model-based clustering approach.

## Methods

### Plant materials

**Figure 1:**
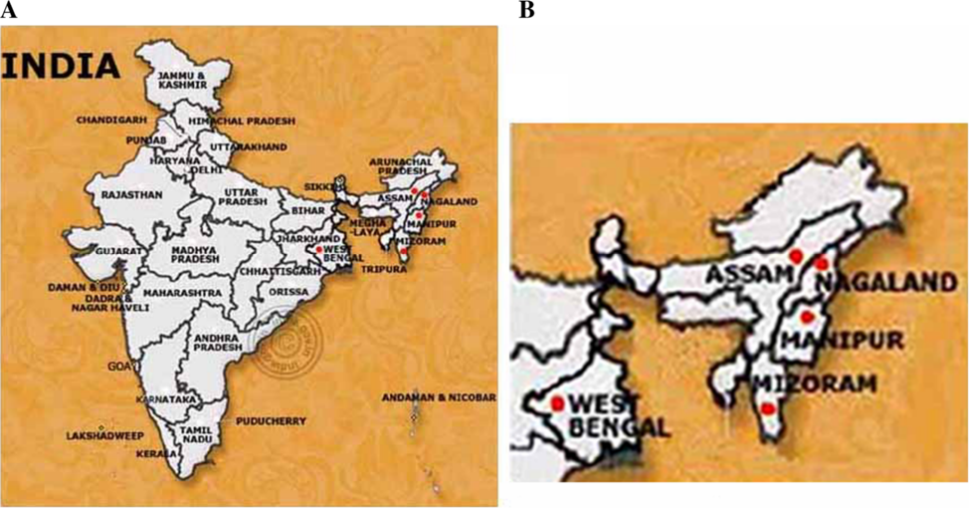
**(A) Political map of India showing the states (marked with red dots) from which rice was collected. (B)** Enlarged view of the states from which rice was collected.

A total of 91 rice germplasms including 83 rice landraces and 8 check germplasms were collected from rice research stations in India. The landraces were divided in 4 categories; namely 26 aromatic accessions from West Bengal, 31 non-aromatic accessions from West Bengal, 26 accessions from the North Eastern States which included 6 aromatic and 6 non aromatic germplasms from Nagaland, 3 non-aromatic germplasms from Mizoram and 1 accession from Manipur and check germplasms included 2 basmati accessions and 6 high yielding varieties. The name, abbreviation source, category, kernel length, kernel shape, amylose content aroma level and inferred group from population structure analysis, of each rice accession are given in Table 1 and the outline maps in Figure 1 show the states from which the collection was made.

**Table 1:** Names, source, category, kernel length, kernel shape, amylose content, aroma and group of the 91 genotypes included in this study

**Genotypes**	**Source**	**Category**	**Kernel length**	**Kernel shape**	**Amylose content**	**Aroma**	Group*
**Accessions of aromatic genotypes from West Bengal**							
Badshahbhog	RRS, Chinsurah	WBA	Small	Medium	Low	Fairly strong	B
Chinikamini 1	RRS, Chinsurah	WBA	Small	Round	Low	Moderately strong	B
Chinikamini 2	RRS, Chinsurah	WBA	Medium	Slender	Intermediate	Fairly strong	C
Danaguri	RRS, Chinsurah	WBA	Small	Medium	Low	Moderate	B
Gobindobhog 1	RRS, Chinsurah	WBA	Small	Medium	Low	Strong	B
Gobindobhog 2	RRS, Sekhampur	WBA	Small	Medium	Low	Strong	B
Gopalbhog	SARF, Kashipur	WBA	Small	Medium	Intermediate	Fairly strong	B
Kalogobindobhog	ATC, Fulia	WBA	Medium	Round	Low	Moderate	D
Kalojira	ATC, Fulia	WBA	Small	Bold	Intermediate	Moderately strong	B
Kalonunia	SARF, Kashipur	WBA	Small	Medium	Intermediate	Fairly strong	B
Kaminibhog	SARF, Kashipur	WBA	Small	Round	Intermediate	Fairly strong	B
Kanakchur	SARF, Kashipur	WBA	Small	Round	Low	Moderate	D
Katarihog	RRS, Chinsurah	WBA	Medium	Medium	Low	Fairly strong	A
Khasdhan	RRS, Chinsurah	WBA	Small	Medium	Low	Moderately strong	B
Lilabati	RRS, Chinsurah	WBA	Small	Bold	Low	Fairly strong	B
Mohanbhog	ATC, Fulia	WBA	Small	Bold	Low	Fairly strong	B
Narayan bhog	ATC, Fulia	WBA	Long	Slender	Low	Moderate	D
Narayan purna	ATC, Fulia	WBA	Small	Round	Low	Fairly strong	D
NC 324	RRS, Chinsurah	WBA	Medium	Medium	Low	Fairly strong	B
NC 365	RRS, Chinsurah	WBA	Medium	Slender	Low	Fairly strong	B
Radhunipgol 1	RRS, Chinsurah	WBA	Small	Bold	Low	Fairly strong	B
Radhunipgol 2	RRS, Chinsurah	WBA	Small	Bold	Low	Fairly strong	B
Radhatilak	RRS, Chinsurah	WBA	Small	Medium	Low	Fairly strong	B
Tulaipanji	RRS, Sekhampur	WBA	Small	Medium	Low	Moderately strong	B
Tulsibhog	RRS, Sekhampur	WBA	Small	Bold	Intermediate	Moderate	B
Tulsimanjari	ATC, Fulia	WBA	Small	Medium	Low	Moderate	B
**Accessions of aromatic genotypes from West Bengal**							
Asitkalma	ATC, Fulia	WBNA	Long	Slender	Intermediate	Nil	D
Bahurupi	ATC Fulia	WBNA	Extra long	Medium	Intermediate	Nil	C
Bangalakshmi	ATC Fulia	WBNA	Extra long	Medium	Intermediate	Nil	C
Bangladeshi patnai	ATC Fulia	WBNA	Extra long	Slender	Intermediate	Nil	C
Bhasamanik	ATC Fulia	WBNA	Extra long	Slender	Intermediate	Nil	D
Chamarmani	ATC, Fulia	WBNA	Extra long	Medium	Intermediate	Nil	C
Dudherswar	SARF, Kashipur	WBNA	Medium	Slender	Intermediate	Nil	C
Hatipanjra	RRS, Chinsurah	WBNA	Medium	Slender	Intermediate	Nil	D
Heerasail	RRS, Chinsurah	WBNA	Long	Medium	Intermediate	Nil	D
Kalisankar	ATC, Fulia	WBNA	Small	Medium	Intermediate	Nil	D
kalopahar	RRS, Chinsurah	WBNA	Extra long	Slender	High	Nil	C
Katki	ATC, Fulia	WBNA	Medium	Medium	Intermediate	Nil	D
Kelas	ATC, Fulia	WBNA	Long	Medium	Intermediate	Nil	D
Kele	ATC, Fulia	WBNA	Long	Medium	Intermediate	Nil	C
Kerala sundari	ATC, Fulia	WBNA	Extra long	Slender	Intermediate	Nil	C
Khandagiri	RRS, Chinsurah	WBNA	Extra long	Slender	High	Nil	C
Lakshmansail	RRS, Chinsurah	WBNA	Extra long	Slender	Intermediate	Nil	D
Latasail	RRS, Chinsurah	WBNA	Extra long	Medium	Intermediate	Nil	D
Madina	SARF, Kashipur	WBNA	Extra long	Slender	Intermediate	Nil	D
Mahsuri	RRS, Chinsurah	WBNA	Extra long	Slender	High	Nil	C
Marichsail	SARF, Kashipur	WBNA	Small	Medium	Low	Nil	D
Metedhan	SARF, Kashipur	WBNA	Extra long	Medium	Intermediate	Nil	C
Mugai	SARF, Kashipur	WBNA	Medium	Medium	Intermediate	Nil	D
Para	ATC, Fulia	WBNA	Medium	Medium	Low	Nil	C
Parbol	ATC, Fulia	WBNA	Extra long	Medium	High	Nil	C
Paizam	ATC, Fulia	WBNA	Extra long	Slender	High	Nil	C
Raghusail	RRS, Chinsurah	WBNA	Medium	Medium	High	Nil	D
Raniakanda	ATC, Fulia	WBNA	Extra long	Slender	Intermediate	Nil	C
Raspanjar	RRS, Chinsurah	WBNA	Long	Medium	Intermediate	Nil	D
Sadashankar	RRS, Chinsurah	WBNA	Extra long	Slender	High	Nil	D
Talmari	RRS, Chinsurah	WBNA	Extra long	Medium	High	Nil	C
**Accessions from North Eastern States**							
Aijong	AAU	NA, ASM	Long	Slender	Intermediate	Nil	D
Biroi dhan	NBPGR, Umiam	NA, ASM	Long	Slender	Low	Nil	A
Bhog joha	NBPGR, Umiam	AR, ASM	Medium	Medium	Intermediate	Faint	A
Bhu	NBPGR, Umiam	NA, MZ	Medium	Medium	Intermediate	Nil	A
Boro Chhaiyamora	AAU	NA, ASM	Long	Slender	Intermediate	Nil	D
Buhrimtui	NBPGR, Umiam	NA, MZ	Medium	Medium	Low	Nil	A
Desi Dhan	NBPGR, Umiam	NA, MZ	Medium	Medium	Intermediate	Nil	A
IC524502	NBPGR, Umiam	NA, NG	Extra long	Medium	Intermediate	Nil	A
IC-524507	NBPGR, Umiam	NA, NG	Extra long	Medium	Intermediate	Nil	A
IC-311005	NBPGR, Umiam	NA, NG	Medium	Medium	Intermediate	Nil	A
IC-311003	NBPGR, Umiam	NA, NG	Extra long	Slender	Low	Nil	A
IC-524526	NBPGR, Umiam	NA, NG	Medium	Medium	Intermediate	Nil	A
IC-524531	NBPGR, Umiam	NA, NG	Extra long	Medium	Low	Nil	A
IC-524528	NBPGR, Umiam	NA, NG	Extra long	Medium	Intermediate	Nil	A
IC-524529	NBPGR, Umiam	NA, NG	Extra long	Medium	Intermediate	Nil	A
IC-311028	NBPGR, Umiam	NA, NG	Extra long	Medium	Intermediate	Nil	A
IC-524530	NBPGR, Umiam	NA, NG	Extra long	Medium	Intermediate	Nil	A
IC360739	NBPGR, Umiam	NA, ASM	Extra long	Medium	Low	Nil	A
Joha	RRS, Chinsurah	AR, ASM	Extra long	Slender	Intermediate	Moderately strong	B
Kala Boro dhan	NBPGR, Umiam	NA, ASM	Medium	Medium	Very Low	Faint	A
Kachalo	NBPGR, Umiam	NA, ASM	Medium	Medium	Intermediate	Nil	A
Kalojeera	AAU	AR, ASM	Small	Slender	Intermediate	Strong	D
Lal Binni	AAU	AR, ASM	Extra long	Slender	Very low	Faint	D
Malsara dhan	NBPGR, Umiam	NA, ASM	Extra long	Medium	Low	Nil	A
Morianghou	NBPGR, Umiam	NA, MN	Medium	Medium	Intermediate	Nil	A
Prasadbhog	AAU	AR, ASM	Long	Slender	Intermediate	Fairly strong	C
**Accessions of check germplasms**							
IR8	RRS, Chinsurah	ICV	Medium	Medium	Low	Nil	C
IR 36	RRS, Chinsurah	ICV	Medium	Slender	Intermediate	Nil	A
IR 64	RRS, Chinsurah	ICV	Extra long	Slender	Intermediate	Nil	C
Khitish	RRS, Chinsurah	HYV	Extra long	Slender	Intermediate	Nil	C
Lal Swarna	SARF, Kashipur	HYV	Medium	Slender	Intermediate	Nil	D
TN 1	RRS, Chinsurah	HYV	Medium	Slender	Intermediate	Nil	C
Pusa Basmati 1	RRS, Chinsurah	IA	Extra long	Slender	Intermediate	Strong	C
Taraori Basmati	ATC, Fulia	IA	Extra long	Slender	Very Low	Strong	B

*Abbreviations: WBA* Aromatic landrace from West Bengal, *WBNA* Non aromatic landrace from West Bengal, *AR ASM* Aromatic landraces from Assam, *NA ASM* Non aromatic landraces from Assam, *NA MN* Non aromatic landraces from Manipur, *NA MZ* Non aromatic landraces from Mizoram, *NA NG* Non aromatic landraces from Nagaland, *ICV* International check variety, *HYV* High yielding variety, *AAU* Assam Agriculture University, *ATC* Agricultural training centre, *RRS* Rice research station, *NBPGR* National Bureau of Plant Genetic Resources, *SARF* State agricultural research farm.

*Groups as was determined by STRUCTURE ANALYSIS.

The classification of kernels different kernel lengths and shapes has been done according to Standard Evaluation System (SES, IRRI) [32]. The data of amylose content and aroma was obtained from previous experiments in our laboratory [33].

### Isolation of genomic DNA and PCR amplification

Three day-old rice seedlings germinated from 10 well developed grains from a single plant of each genotype were used for genomic DNA isolation according to the method of Walbot [34]. PCR amplification of this DNA was done with 23 pairs of SSR markers. The name, motif, chromosomal location, annealing temperature of the markers, their associated phenotypic trait and corresponding references are given in Table 2. DNA amplification was carried out in 25 μl volumes in a MJR thermal cycler (USA). Each reaction mixture contained 1 μl of genomic DNA (100 ng), 0.5 μl of each primers (at a concentration of 10 pmole/μl), 2.5 μl of 10× PCR buffer, 0.75 μl of 50 mM MgCl_2_, 0.25 μl of 2.5 mM dNTP mixture, 0.2 μl (1 unit) of 5 unit/μl Taq DNA polymerase and 19.3 μl of PCR-grade water. The temperature profile of the first PCR cycle was 97°C for 5 mins, 55-60°C (as necessary in accordance to Table 2) for 2 min; followed by 35 cycles of 1 min at 95°C, 1 min at 55-60°C and 2 min at 72°C. The final extension was at 72°C for 10 min.

**Table 2:** Name, motif, chromosomal location, annealing temperature and associated phenotypic trait of each SSR marker used for this study

**Locus**	**Motif**	Chr*	T**	**Associated phenotypic trait**	**Reference**
RM 42	(GA)6	8	65	LAC,CKE,AROMA	Bisaws 2004, Shobha Rani 2006
RM 44	(GA)16	8	55	LAC, L/B AC,CKE	Biswas 2004, Shobha Rani 2006
RM72	(TAT)5C(ATT)15	8	55	Sheath blight resistance	Eizenga *et al* 2006
RM 80	(CTT)20	8	65	LAC,AROMA	Bisaws 2004, Shobha Rani 2006
RM 112	(GAA)5	2	55	GL,GB,G L/B,KL,KB,K L/B	Biswas 2004, Shobha Rani 2006, Bai *et al*., 2010, Lin *et al*., 2007
RM 149	(AT)10	8	59	HSP 80 gene	Breusegem *et al* 1994, Eizenga *et al*., 2006
RM 152	(GGC)10	8	60	GL,GB,G L/B,KL,KB,K L/B	Biswas 2004, Shobha Rani 2006
RM 182	(AT)16	7	59	Beta amylase gene	Chen, 1993
RM207	(GA)25	2	65	GL,AROMA	Bisaws 2004, Shobha Rani 2006
RM 210	(GA)23	8	55	AROMA	Biswas 2004,
RM 218	(GA)24	3	55	GL	Biswas 2004, Shobha Rani 2006
RM 223	(GA)25	8	55	LAC, L/B AC,CKE	Biswas 2004, Shobha Rani 2006
RM 250	(CT)17	2	60	GL,GB,G L/B,KL,KB,K L/B	Biswas 2004, Shobha Rani 2006
RM 251	(CT)29	3	55	Sheath blight resistance	Eizenga *et al*., 2006, Ahmadi and Fotokian 2011
RM 282	(GA)15	3	59	Blast resistance	Eizenga *et al*., 2006, Ahmadi and Fotokian 2011
RM 284	(GA)8	8	55	LAC, L/B AC,CKE	Biswas 2004, Shobha Rani 2006
RM 310	(GT)19	8	55	GL,GB,G L/B,KL,KB,K L/B	Biswas 2004, Shobha Rani 2006
RM 337	(CTT)4-19-(CTT)8	8	59	AROMA	Biswas 2004, Shobha Rani 2006
RM 339	(CTT)8CCT9CCT)5	8	59	LAC, L/B AC,CKE	Biswas 2004, Shobha Rani 2006
RM 341	(CTT)20	2	55	Sheath blight resistance	Eizenga *et al*., 2006
RM 505	(CT)12	7	55	GL,GB,G L/B,KL,KB,K L/B	Biswas 2004, Shobha Rani 2006
RM 530	(GA)23	2	59	GL,GB,G L/B,KL,KB,K L/B	Biswas 2004, Shobha Rani 2006, Bai *et al*., 2010, *Lin et al*., 2007
RM 569	(CT)16	3	59	GL,GB,G L/B,KL,KB,K L/B	Biswas 2004, Shobha Rani 2006

Chr - Rice chromosome T - Annealing temperature.

### Polyacrylamide Gel electrophoresis

The PCR products were resolved in native polyacrylamide gel electrophoresis (PAGE) according to Sambrook *et al*. [35] in 6% gel in vertical electrophoresis tank (gel size of 16 cm × 14 cm, Biotech, India) with Tris-Acetate-EDTA buffer at 150 V. The gel was stained with ethidium bromide (5 μg of EtBr in 200 ml of Tris-Borate-EDTA buffer) washed twice with distilled water and analyzed in Gel Documentation System (Biorad, USA).

### Allele scoring

A cluster of two to five discrete bands (stutter) was apparent in the stained gels for most of the markers. The size (in nucleotides) of the most intensely amplified band for each microsatellite marker was determined using the Quantity One software (Biorad, USA), based on the migration of the band relative to standard molecular weight size markers (100 bp DNA ladder SibEnzyme) [36]. IR36 was used as a molecular weight reference in each gel because a sequence-based estimate of allele size in this germplasm is available, as described in Panaud et al., [11]. The band with the lowest molecular weight for each SSR marker was assigned allele number 1 and the progressively heavier bands were assigned incrementally. For the individual markers, the presence of an allele in each of the germplasms was recorded as “1” and the absence of an allele was denoted as “0” [36]. A genotype was assigned a null allele for an SSR locus whenever an amplification product(s) was not detected for the particular genotype × marker combination [37]. When an allele was found in less than 5% of the germplasms under study, it was designated as rare [38].

### Genetic diversity analysis using SSR profiles and bootstrap analysis

A 1/0 matrix was constructed for each marker using the information of presence or absence of alleles. The resultant matrix was used to calculate genetic similarities among the accessions according to Jaccard’s coefficient [39] using NTSYS-pc software package (version 2.02e) [40]. Using pairwise similarity matrix of Jaccard’s coefficient, a phylogenetic tree was constructed by the Unweighted Pair-Group Method of Arithmetic average (UPGMA) and Neighbor-Joining (NJoin) module of the NTSYS-pc. Support for clusters was evaluated by bootstrap analysis using WinBoot software [41] through generating 1,000 samples by re-sampling with replacement of characters with in the combined 1/0 data matrix.

### Calculation of polymorphism information content

The polymorphism information content (PIC) value modified by Anderson et al., [42] for self pollinated species was used to calculate the PIC value. The formula is as follows:

$${\mathrm{PIC}}_{i}=1–{\sum {}^{n}}_{i=1}{\;P}^{2}{}_{\mathrm{ij}},$$

where Pij is the frequency of the j^th^ allele for the i^th^, SSLP marker.

### Population structure analysis

The association mapping population was analyzed for possible population structure with the model-based program Structure 2.2 [43] for the 91 rice accessions using a length of burn-in period and the number of iterations set at 10000 and a model allowing for admixture and correlated allele frequencies. At least ten runs of Structure were performed by setting the number of sub-populations (K) from K = 2 to K = 10. Pair-wise FST values were estimated among four sub-populations using Arlequin [44].

## Results

### Analysis of SSR profiles

#### Number of alleles

Table 3 summarizes the analysis of SSR profiles of the 91 rice accessions using 23 SSR markers. The reference molecular weight derived from the standard accession IR-36, for each marker is also showed in Table 3. All the markers showed polymorphism and a total of 182 alleles were identified. The number of alleles ranged from 2 in RM112 to 13 in RM 149. The average number of alleles was 7.9 alleles per locus. Categorically, the average number of alleles for aromatic rice accessions from West Bengal was 5.13 alleles /locus. The SSR profiles of the markers RM42, RM210 and RM530 were the least informative with 3 alleles each, while the most informative marker was RM341 producing 8 alleles. The average number of alleles for the non-aromatic rice accessions from West Bengal was 6.69 alleles /locus. The number of alleles ranged from 4 in the SSR profiles of the markers RM112, RM282 and RM341 to 9 for the markers RM 149, RM223 and RM250. For the germplasm accessions from the North East Indian States, the average number of alleles was 4.91 alleles /locus. In this group only two alleles were produced by the markers RM112 and RM505, while the highest of 8 alleles was generated by RM149. For the check genotypes the average number of alleles was 2.87 alleles /locus with just 1 allele produced by RM505 and the highest of 4 alleles generated by the markers RM80, RM152, RM210 and RM337.

**Table 3:** Minimum and maximum molecular weight among the alleles, rare alleles (R), null alleles (N) and PIC values for each marker

**Marker**	**min MW**	**max MW**	**IR 36 MW**	**R**	**N**	**Number of alleles**					**PIC values**				
						**Total**	**WBA**	**WBNA**	**Check**	**NE**	**Total**	**WBA**	**WBNA**	**Check**	**NE**
RM 42	114.39772	189.36196	166	2	4	9	3	7	3	8	0.8114962	0.5535714	0.830385	0.625	0.8343195
RM 44	89.648764	109.72365	99	1	1	6	5	6	3	4	0.7793745	0.5765306	0.817898	0.71875	0.6272189
RM 72	137.82168	182.30073	166	2	1	7	5	6	3	5	0.7453206	0.5433673	0.8022893	0.671875	0.3934911
RM 80	113.39867	178.60759	142	1	5	7	6	7	4	3	0.7565511	0.7627551	0.8605619	0.65625	0.6005917
RM 112	119.83476	139.12983	128	1	0	4	4	4	3	2	0.534235	0.5331633	0.5681582	0.40625	0.204142
RM 149	169.15164	277.81621	253	3	0	13	6	9	2	11	0.8701848	0.7295918	0.9646202	0.703125	0.8816568
RM 152	124.85913	159.70665	151	1	1	5	5	6	4	4	0.7045043	0.6836735	0.7658689	0.65625	0.3313609
RM 182	307.94713	372.9556	346	2	0	9	7	7	3	4	0.8246589	0.8010204	0.8220604	0.75	0.693787
RM 207	64.904795	178.32084	118	4	4	9	6	8	2	7	0.7722497	0.6887755	0.7793965	0.375	0.8239645
RM 210	110.11641	218.32062	140	3	2	9	3	9	4	4	0.787103	0.6607143	0.9927159	0.5625	0.5325444
RM 218	112.20192	161.50204	148	2	1	8	5	7	3	5	0.8134283	0.6479592	0.7887617	0.40625	0.7869822
RM 223	139.79416	192.30402	165	3	1	9	7	9	3	5	0.8284024	0.7653061	0.8647242	0.65625	0.6390533
RM 250	121.09209	174.86578	153	6	2	9	4	9	3	4	0.6330153	0.2576531	0.867846	0.53125	0.5946746
RM 251	104.74847	178.47583	147	1	1	8	5	8	3	5	0.779133	0.625	0.9354839	0.8125	0.6065089
RM 282	122.1457	146.14296	136	2	2	8	4	4	2	6	0.8236928	0.7270408	0.7055151	0.421875	0.6538462
RM 284	129.65555	161.73167	141	3	0	7	5	6	2	5	0.7383166	0.6505102	0.6930281	0.21875	0.535503
RM 310	58.878678	126.12462	105	2	1	9	6	7	3	5	0.8030431	0.7142857	0.8158169	0.78125	0.6745562
RM 337	144.766	383.20582	192	2	0	8	5	7	4	6	0.7913295	0.7385204	0.8896982	0.71875	0.6449704
RM 339	136.11499	194.5526	148	1	0	8	5	5	3	4	0.7153725	0.6938776	0.5296566	0.40625	0.4763314
RM 341	124.84082	251.62117	172	2	0	8	8	4	3	4	0.7204444	0.8545918	0.6181061	0.59375	0.5680473
RM 505	173.52414	249.29178	199	3	0	7	5	6	1	2	0.484241	0.6964286	0.9677419	0	0.0739645
RM 530	137.17836	177.19601	161	2	1	7	3	6	2	4	0.7032967	0.3137755	0.7700312	0.375	0.3920118
RM 569	136.86345	194.98306	175	2	0	8	6	7	3	6	0.7557058	0.7882653	0.760666	0.53125	0.6449704
Sum				51	27	182	118	154	66	113	17.1751	15.006378	18.41103	12.578125	13.214497
Average				2.21	1.17	7.91	5.13	6.69	2.87	4.91	0.7467435	0.6524512	0.8004796	0.546875	0.5745433

*Abbreviations: minMW* Minimum molecular weight of the alleles in that locus, *max MW* Maximum molecular weight of the alleles in that locus, *WBA* Aromatic landrace from West Bengal, *WBNA* Non aromatic landrace from West Bengal, *NE* Landraces from North Eastern States, *Check* Check genotypes, *IR36 MW* Molecular weight of the allele obtained from the reference genotype IR 36.

#### PIC values

For all the accessions the PIC values, which denote allelic diversity and frequency, had an average value of 0.747 per marker. The range of PIC value was 0.484 in RM 505 to 0.871 in RM 149. Categorically, average PIC value for aromatic rice from West Bengal was 0.652 per marker with a range of 0.258 in RM 250 to 0.855 in RM 341. For the non aromatic germplasm accessions of West Bengal, the average PIC value was 0.801 per marker and range of PIC value was 0.528 in RM 339 to 0.993 in RM210. The North East Indian set of rice had an average PIC value of 0.575 per marker. The range of PIC value for them was 0.0739 in RM505 to 0.882 in RM 149. For the check rice germplasms the average PIC value was 0.547 per marker. The range of PIC value was 0.0 in RM 505 to 0.812 in RM 251.

#### Rare alleles

A total of 51 rare alleles were identified from the 23 polymorphic loci. The highest number of rare alleles (6) was generated by the marker RM250 followed by RM207 (4 rare allele) and RM149, RM210, RM223, RM284 and RM505 generated 3 rare alleles each. Among the landraces, Kelas a non aromatic landrace from West Bengal had 7 rare alleles which is the maximum number in this set of rice accessions. Bangalaxmi and Bangladeshi Patnai, both non aromatic landraces from West Bengal have 5 rare alleles. Among the aromatic rice, the landrace Tulsimanjari from West Bengal, and the check Taraori Basmati had 4 rare alleles each. Chinikamini 2 and Narayanpurna, both aromatic landraces from West Bengal had 3 rare alleles each. Among the landraces from North East Indian States, Biroi dhan had 4 rare alleles followed by Aijung, Kalo Jeera, IC524502 and Morianghou with 3 rare alleles each.

#### Null alleles

Twenty seven null alleles were detected from 14 out of the 23 polymorphic loci. The highest number of null alleles (5 alleles) was generated by the marker RM80, followed by RM42 and RM207 with 4 null alleles each. Among the landraces, Kelas, a non aromatic landrace from West Bengal, had 4 null alleles, which is the highest number in this set of rice accessions. Bangalaxmi and Raghusail, both non aromatic landraces from West Bengal, and IC524531 from Nagaland had 3 null alleles each.

### Clustering of the rice genotypes

**Figure 2:**
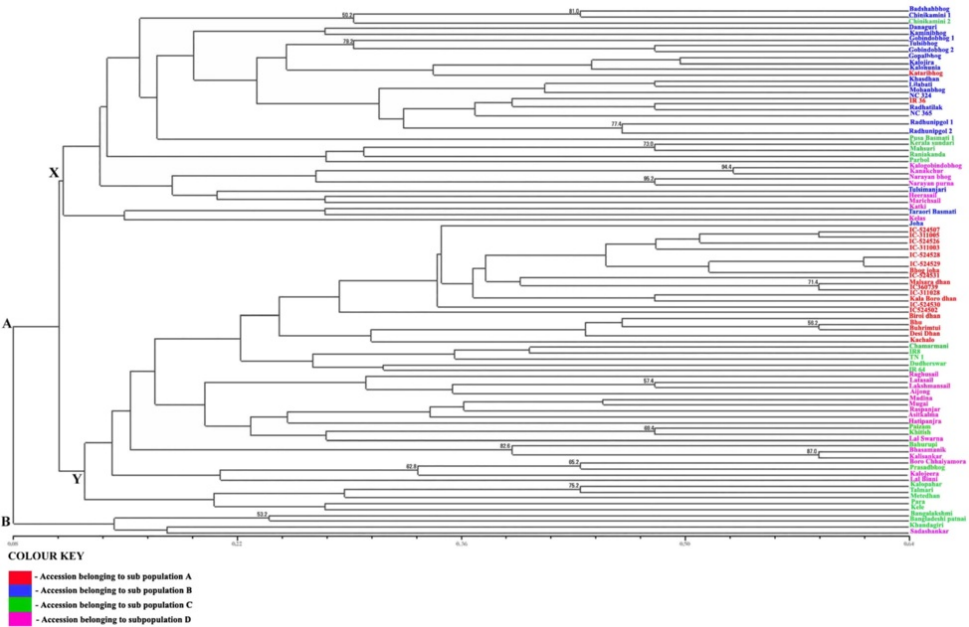
Dendrogram of 91 rice genotypes based on Jaccard’s genetic similarity coefficients.

The dendrogram given in Figure 2 was made from genetic similarity values. The strength of dendrogram nodes was estimated with a bootstrap analysis using 1000 permutations. The similarity among the rice accessions ranged from 8% to 64%. At 8% level of similarity the dendrogram showed 2 clusters “A” and “B” with additional sub clusters within each. The cluster B contained only four rice accessions, Bangalaxmi, Bangladeshi Patnai, Khandagiri and Sada Sankar; all non-aromatic landraces from West Bengal. The rest 87 accessions are grouped in cluster “A”. At 10.8% level of similarity cluster “A” divided into two sub clusters, of which one, cluster “X”, consisted predominantly of aromatic rice accessions from West Bengal and the other, cluster “Y”, consisted predominantly of accessions from North Eastern States. The non aromatic rice accessions from West Bengal and the check genotypes were interspersed within the two predominant clusters.

The aromatic cluster “X” consisted of a total of 37 rice genotypes. The set included 27 aromatic landraces from West Bengal, 8 non aromatic landraces from West Bengal, and 3 check genotypes (IR36, Pusa Basmati 1 and Taraori Basmati). Within this cluster Kalogobindobhog and Kanakchur were 53% similar and the two accessions of Radhunipagol, one each from repositories at Chinsurah and Phulia were 64% similar between themselves. The cluster was subdivided into two prominent sub clusters at about 11% level of similarity after which it segregated repeatedly to form sub clusters at progressively higher level of similarity until 64% level of similarity beyond which it segregated no more. Super imposition of the results of population structure analysis showed that the whole of sub population B was included in this cluster along with 8 members from sub population D. Six members from sub population C and 2 from sub population A were also included.

The predominant North Eastern cluster “Y” consisted of 50 different rice accessions out of which 12 were from Assam, 10 accessions from Nagaland, 1 from Manipur, 3 from Mizoram, 19 non-aromatic landraces from West Bengal and 5 check genotypes. The cluster included the 2 accessions having the lowest amylose content (Lal Binni and Kalo Boro Dhan from Assam). The most similar accessions included IC524528 and IC524529 from Nagaland (64% similar), and Bhu and Buhrimtui from Mizoram (58.4% similar). Accessions of sub population A formed the majority in this cluster followed by accessions of sub population D and C. At 13.6% level of similarity a sub cluster consisting of five non-aromatic landraces from West Bengal separated out. The genotypes in this group Kalopahar, Metedhan, Talmari, Kele and Para, had extra long to medium kernel length, medium kernel length and intermediate amylose content and were included in sub population C. There were no segregations beyond 64% level of similarity.

### Population structure analysis

**Figure 3:**
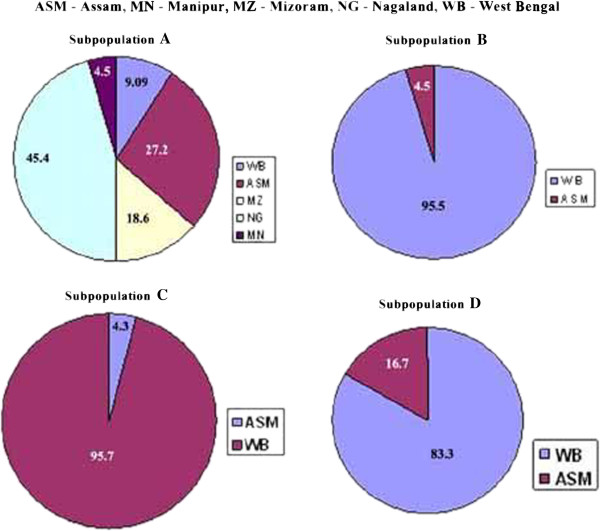
Pie diagram showing frequencies of individuals belonging to the four (A-D) structure group based on sampling area.

The inferred population structure is given in Figure 3. Admixture model-based simulations were carried out by varying K from 2 to 10 with 10 iterations using all 91 genotypes which showed evident knees at K = 4 (Additional file 1: Figure S1 and Additional file 2: Figure S2) and were assigned to the corresponding A-D sub-populations representing 23.0% of A (21), 25.3% of B (23), 25.3% of C (23) and 26.4% of D (24) used for the structure analysis. Genetic variation with subpopulations was tested using Fst statistics. The four sub-populations (A-D) had an Fst value of 0.398, 0.364, 0.206 and 0.281, respectively, with an average value 0.312, indicating moderate population structure (Figure 3). These are population specific Fst value (not pair-wise Fst values between subpopulation) for 4 sub-populations (A-D) calculated using STRUCTURE software during construction of population structure. Based on sampling area frequencies of individuals belonging to each Structure group a pie diagram is given in Figure 3. Pair-wise Fst among the four sub-populations and their level of significance are given in Table 4.

**Table 4:** Pair-wise FST estimation among four sub-populations estimated using Arlequin

**Populations**	**Populations groups**			
	**A**	**B**	**C**	**D**
**A**	**-**	**0.20**	**0.14**	**0.16**
**B**	0.22**	**-**	**0.15**	**0.14**
**C**	0.19*	**0.15**	**-**	0.10*
**D**	**0.17**	**0.15**	0.08**	**-**

*Significance at P <0.05 at 1,000 permutations.

** Significance at P <0.01 at 1,000 permutations.

The subpopulation A consisted of 22 accessions out of which 20 were from the North Easetrn States along with Kataribhog – an aromatic accession from West Bengal and IR36 – a high yielding check accession. They have extra long to medium grain length, predominantly medium kernel shape and intermediate amylose content. However the accession (Kalo Boro Dhan of Assam) with lowest amylose content was included in this group. The average number of alleles was 5.39 alleles / SSR marker. The prominent accessions having rare alleles were Biroi Dhan with 4, and Aijung, IC524502 and Morianghou with 3 rare alleles each.

The subpopulation B consisted of 22 aromatic accessions out of which 20 were from West Bengal, along with Joha from Assam and Taraori Basmati, a traditional basmati used as check in this study. This group was dominated by small kernel length, mostly medium kernel shape and low amylose content. The average number of alleles was 5.91 alleles / SSR marker. The prominent accessions having rare alleles were Tulsimanjari and Taraori Basmati, each with 4 rare alleles. There were 5 null alleles in this subpopulation wherein the accessions Danaguri and Lilabati have 2 and Badshahbhog with 1 null allele.

The subpopulation C consisted of 23 accessions which included 16 were non aromatic and 1 aromatic accessions from West Bengal, along with Prasasdbhog from Assam and 5 accessions used as check in this study. This group was dominated by extra long kernel length, medium kernel shape and intermediate to high amylose content. The average number of alleles was 8.22 alleles / SSR marker. The prominent accessions with rare alleles were Bangalaxmi and Bangladeshi Patnai, each with 5 rare alleles. There were 8 null alleles in this subpopulation wherein the accessions Bangalaxmi had 3 Chamormoni and Metedhan had 2 and Para had 1 null allele.

The subpopulation D consisted of 24 accessions which included 16 were non aromatic and 4 aromatic accessions from West Bengal, along with 4 accessions from Assam. This group was characterized by small to extra long kernel length, round to slender kernel shape and very low to high amylose content. The average number of alleles was 7.35 alleles / SSR marker. The prominent accessions with rare alleles were the non aromatic accession from West Bengal, Kelas with 7 and Heerasail and Sada Sankar with 3 rare alleles each. There were 7 null alleles in this subpopulation wherein the accessions Kelas had 4 and Raghusail had 3 null alleles.

## Discussion

This study calculates the genetic distances among accessions of 83 landraces from West Bengal and the North Eastern States and 8 check accessions using 23 SSR markers and examines the population structure among the accessions using model-based clustering approach. The set included both aromatic and non aromatic accessions. The 23 SSR markers used for this study were mapped previously [45]. All the SSR markers used for this study revealed a clear and consistent amplification profile. The results are consistent with published reports on microsatellite frequency in the rice genome [46]. Stutter bands, which are minor products amplified in PCR that have lower intensity than the main allele and normally lacks or has extra repeat units [37] were also present in the profiles of most of the markers used. Null alleles were present probably due to mutations in the binding region of one or both of the microsatellite primers, thereby inhibiting primer annealing [37]. The number of alleles ranged from 2 in RM112 to 13 in RM 149 and the average was 7.9 alleles / locus. This number is higher than that reported for average number of alleles by Cho (5.5 alleles/locus) [36] or Yu *et al*., (6.3 alleles/locus) [46]. This is also smaller than the study by Yang *et al*., [15] which reported 9.3 alleles/ locus. However the average number of allele per locus for this study is comparable to reports of 7.8 alleles /locus by Jain et al., [47]; as recorded from a set of Indian aromatic and quality rice accessions. From the PIC values it is evident that within the landraces the allelic diversity is the highest among the non aromatic germplasm accessions from West Bengal followed by the aromatic accessions from the same state. The allelic diversity of the accessions from the North Eastern States stands third for this experimental set of rice accessions.

Most of the markers were selected from chromosome 8 and two important traits of aromatic and basmati rice, aroma and cooked kernel elongation ratio [42], had been mapped earlier using RFLP markers to chromosome eight. In the international market there is a huge demand for long, slender rice kernels which elongate to twice or more the original length. One of the aims of the study was to find out whether the chosen set of rice could be segregated into various categories according to their length and shape of their kernels. The clustering of the rice genotypes according to the SSR profiles however was not able to distinctly classify the set into small, medium, long or extra long length groups. This discrepancy was perhaps due to the fact that the loci involved in this study reveal only a small part of the genotype/phenotypes association of otherwise complex traits like grain and kernel length and shape, which are actually attributed to multiple loci with small effect [24,25].

The analysis of the SSR profiles of all the aromatic landraces from West Bengal and Assam and check basmati rice revealed that there were 13 rare alleles each within the set of 27 aromatic landraces of West Bengal and 6 aromatic landraces Assam. The two check basmati genotypes had 5 rare alleles. Clearly the diversity present in the Assam landraces are of a higher magnitude. Also in the dendrogram in Figure 2 Assam landraces were grouped in a separate cluster from the rest of the aromatic landraces and Basmati rice indicating a difference in their origin. Inclusion of a substantial number of Basmati genotypes would perhaps given rise to a separate cluster for them since the distinctiveness of Basmati from indigenous aromatic rice has already been proved by previous workers [48-51].

Of the total number of different alleles identified, the highest (154 alleles) was found in the non aromatic landraces from West Bengal. That set also had the highest average PIC value/marker. One of the non aromatic genotypes Kelas had 7 rare and 4 null alleles. In addition the accessions Bangalaxmi and Bangladeshi Patnai, had 5 rare alleles each. This category of rice has been subjected to lesser human selection as compared to the aromatic genotypes. The aromatic genotypes of West Bengal have been specifically selected for traits like slender kernels, non sticky cooked kernels, shorter cooking time and strong aroma thereby narrowing their genetic base. Their end use is also limited to making desserts, as quality table rice and as offerings in Hindu ceremonies. The non aromatic rice genotypes on the other hand has undergone lesser selection pressure and are used for a variety of purposes like making of table rice, puffed rice, popped rice, beaten rice, rice flakes etc. Some of them like Kabirajsail (not included in this study) are reported to have medicinal values and some others like Raghusail, Bhasamanik and Kelas are reported to have resistance against bacterial leaf blight [52].

From the total number of alleles (113 alleles) and the average PIC values (0.5745/marker) it is evident that the genetic diversity in the genotypes from the North Eastern States were far lesser than the diversity of genotypes from West Bengal. Interestingly within the 26 North Eastern rice genotypes, the 12 genotypes from Assam had 94 alleles and an average PIC value of 0.6581/marker. The rest of the North East accessions, of which majority were from Nagaland, had 71 alleles and an average PIC value of 0.4214/marker. The topography of Assam is characterized by both hills and plains with an altitude ranging from 43 m –1736 m and a climate type from tropical to subtropical. The hilly areas of Karbi Anglong and North Cachar Hills are dominated by “jhoom” (slash and burn method) cultivation while the plains of Assam are dominated by lowland and deep water rice cultivation [6]. On the other hand the states of Manipur, Mizoram and Nagaland are characterized by a subtropical to temperate type of climate, with an average land elevation ranging from 235 m to 2745 m and the rice cultivation is mostly upland type [6]. The more diverse nature of the Assamese landraces may be a reflection of the prevalent diverse agroclimatic conditions of the State. However, further collection and characterization of more landraces are required to establish this association.

The population structure analysis revealed 4 subpopulations A, B, C and D out of which the majority of accessions were included in subpopulation D. The groupings of accessions obtained using structure analysis are well in agreement with the distance-based clustering. In the dendrogram the accessions of subpopulations “A” and “B” were far less scattered than the accessions of subpopulations C and D. The kernel length, kernel shape, amylose content and aroma levels of the accessions of subpopulation A were distinctly different from that of subpopulation B. Subpopulation “A” consisted mainly of accessions from the North Eastern States and subpopulation “B” consisted of aromatic accession from West Bengal. Hence some kind of geographical grouping was evident between these two subpopulations. However, in subpopulations C and D, which consisted mainly of non aromatic accessions from West Bengal and the check genotypes with more or less similar kernel morphology; no geographical grouping was obvious.

## Conclusion

Each of the accessions could be identified unequivocally by the SSR profiles. There are obvious differences between the aromatic and non aromatic landraces and the check genotypes, which are evident in the dendrogram. Genetically the non-aromatic landraces from West Bengal were most diverse followed by the aromatic landraces from the same state. The North Eastern accessions ranked third. Further, grouping of accessions based on their agronomic traits may serve as a resource for future studies, leading to the improvement of rice. Although the Basmati rice fetches a premium in the international market due to consumer quality preference of the West, it is a traditional observation that the aromatic landraces are more versatile. They are more soothing to the palate and can be used for a variety of cuisine starting from table rice to desserts, from offerings in various from religious ceremonies to diet for the convalescent and for making popped rice. Basmati accessions on the other hand can be used only for making certain rice delicacies and can never be used for other purposes. The landraces, whether aromatic or not, were confirmed to be adapted to the agro climatic conditions of their respective place of cultivation. Interestingly, Tulaipanji, an aromatic accession originally cultivated in the cooler northern districts of the state of West Bengal, India, was found to lose its aroma when cultivated in the warmer southern districts [52]. Some of these aromatic landraces are also resistant to biotic stress, for example Kataribhog is resistant to rice Tungro virus [52]. The quality of straw produced by the landraces is more suitable for thatching huts [52]. Moreover, the landraces are the means of sustenance for the marginal and impoverished tribal farmers. In-situ preservation of the landraces found in the biodiversity hotspots is also a means of protection of the culture, heritage and socio economic structure of the farmers’ population of those places.

## Competing interests

The authors declare that they have no competing of interests.

## Authors’ contributions

BD did all the experiments pertaining to DNA extraction, PCR, PAGE, collected data and was involved in data analysis and drafting of the manuscript. SS procured the rice accessions from various repositories of the North Eastern States, did some of the experimentation pertaining to PCR and PAGE and helped with data collection and analysis. BR helped with maintenance of the rice accessions. MG procured the rice accessions from various repositories of West Bengal and helped with the experimentation. MP and SKP did the bootstrap analysis and population structure analysis and analysis with arlequin. TKG was involved with the conception of the work and gave the final approval to the version of the manuscript that is being sent for consideration for publication. All authors read and approved the final manuscript.

## Supplementary Material

Additional file 1: Figure S1 Inferred population structure. K varying from 2 to 10 with 10 iterations. Click here for file

Additional file 2: Figure S2 Optimization of K (number of populations) value in population genetic structure using log probability data Ln (K) as function based on Island model (IM) of microsatellite markers (Evanno et al. 2005). The population structure among rice genotypes was found most suitable at K = 4 with Ln (K) value of -10206. Click here for file
