# Supplementary figures and images for: Genetic diversity and population structure of rice landraces from Eastern and North Eastern States of India
Source: BMC Genet. 2013 Aug 15;14:71. doi: 10.1186/1471-2156-14-71 (PMC3765237; doi:10.1186/1471-2156-14-71)

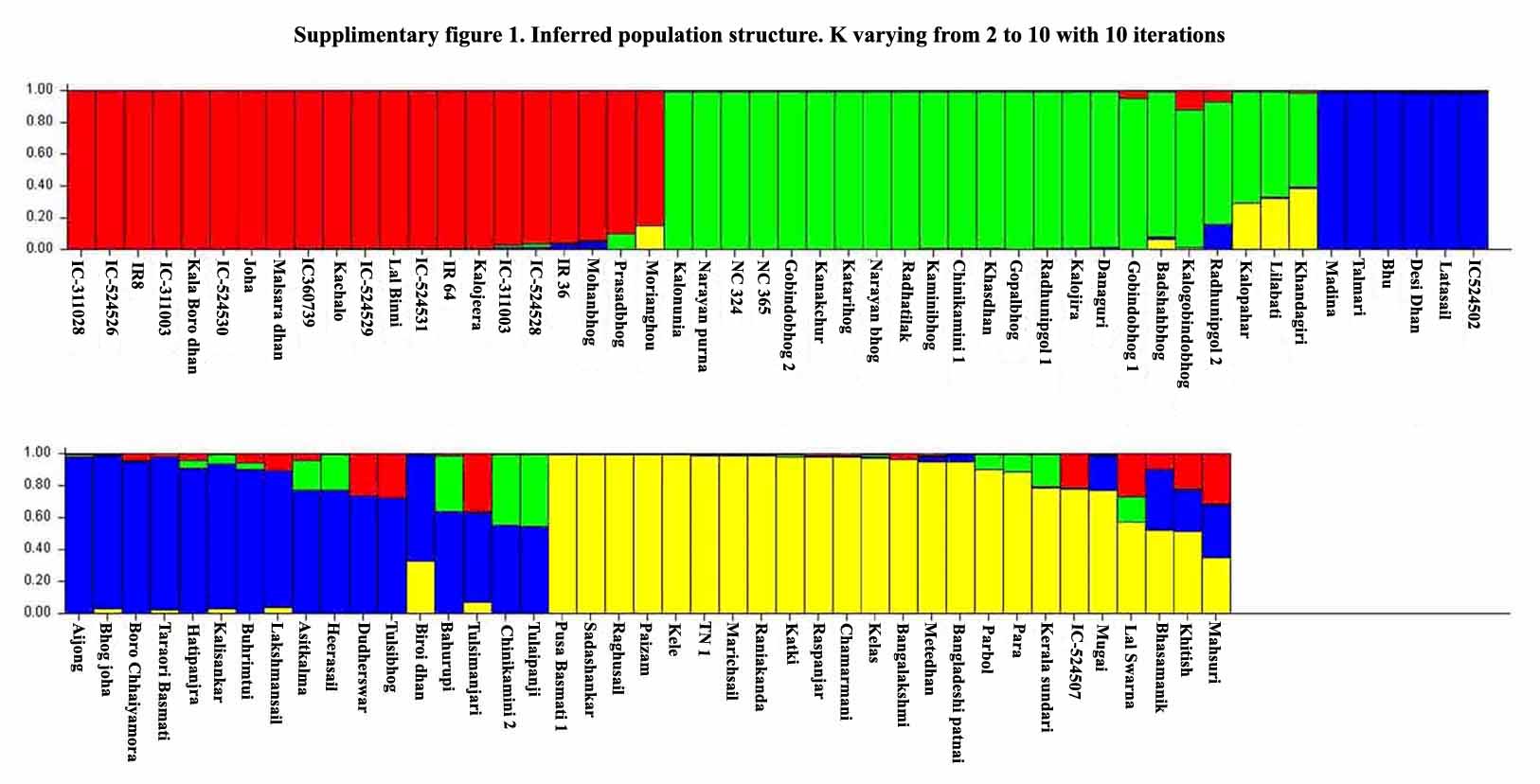

Supplement: Additional file 1: Figure S1 — Inferred population structure. K varying from 2 to 10 with 10 iterations. [file 1471-2156-14-71-S1.jpeg]

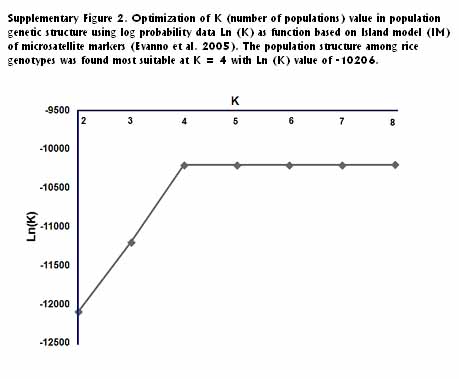

Supplement: Additional file 2: Figure S2 — Optimization of K (number of populations) value in population genetic structure using log probability data Ln (K) as function based on Island model (IM) of microsatellite markers (Evanno et al. 2005). The population structure among rice genotypes was found most suitable at K = 4 with Ln (K) value of -10206. [file 1471-2156-14-71-S2.jpeg]
